# Urinary neutrophil gelatinase-associated lipocalin is associated with heavy metal exposure in welding workers

**DOI:** 10.1038/srep18048

**Published:** 2015-12-17

**Authors:** Kai-Jen Chuang, Chih-Hong Pan, Chien-Ling Su, Ching-Huang Lai, Wen-Yi Lin, Chih-Ming Ma, Shu-Chuan Ho, Mauo-Ying Bien, Cheng-Hsien Chen, Hsiao-Chi Chuang

**Affiliations:** 1 School of Public Health, College of Public Health and Nutrition, Taipei Medical University, Taipei, Taiwan; 2 Department of Public Health, School of Medicine, College of Medicine, Taipei Medical University, Taipei, Taiwan; 3 Institute of Labor, Occupational Safety and Health, Ministry of Labor, New Taipei City, Taiwan; 4 School of Public Health, National Defense Medical Center, Taipei, Taiwan; 5 Division of Pulmonary Medicine, Department of Internal Medicine, Shuang Ho Hospital, Taipei Medical University, New Taipei City, Taiwan; 6 School of Respiratory Therapy, College of Medicine, Taipei Medical University, Taipei, Taiwan; 7 Center of Environmental and Occupational Medicine, Kaohsiung Municipal Hsiaokang Hospital, Kaohsiung, Taiwan; 8 Department of Cosmetic Application and Management, St. Mary’s Junior College of Medicine, Nursing and Management, Yilan County, Taiwan; 9 Division of Pulmonary Medicine, Department of Internal Medicine, Taipei Medical University Hospital, Taipei, Taiwan; 10 Division of Nephrology, Department of Internal Medicine, Wan Fang Hospital, Taipei Medical University, Taipei, Taiwan; 11 Division of Nephrology, Department of Internal Medicine, Shuang Ho Hospital, Taipei Medical University, New Taipei City, Taiwan

## Abstract

Metals cause nephrotoxicity with acute and/or chronic exposure; however, few epidemiological studies have examined impacts of exposure to metal fumes on renal injury in welding workers. In total, 66 welding workers and 12 office workers were recruited from a shipyard located in southern Taiwan. Urine samples from each subject were collected at the beginning (baseline) and end of the work week (1-week exposure). Personal exposure to PM_2.5_ was measured. The 8-h mean PM_2.5_ was 50.3 μg/m^3^ for welding workers and 27.4 μg/m^3^ for office workers. iTRAQs coupled with LC-MS/MS were used to discover the pathways in response to welding PM_2.5_ in the urine, suggesting that extracellular matrix (ECM)-receptor interactions are a critical mechanism. ECM-receptor interaction-related biomarkers for renal injury, kidney injury molecule (KIM)-1 and neutrophil gelatinase-associated lipocalin (NGAL), were significantly elevated in welding workers post-exposure, as well as were urinary Al, Cr, Mn, Fe, Co, and Ni levels. NGAL was more significantly associated with Al (r = 0.737, p < 0.001), Cr (r = 0.705, p < 0.001), Fe (r = 0.709, p < 0.001), and Ni (r = 0.657, p < 0.001) than was KIM-1, suggesting that NGAL may be a urinary biomarker for welding PM_2.5_ exposure. Nephrotoxicity (e.g., renal tubular injury) may be an emerging concern in occupational health.

Welding fumes consist of a complex mixture of magnetite-like particles that are potentially harmful to human health^1^. Symptoms such as occupational asthma, bronchitis^2^, metal fume fever^3^, and cardiovascular effects^4^ were previously reported. Notably, increasing reports have shown an association between metal fume exposure and nephrotoxicity. For example, the urinary renal tubular biomarker, β_2_-microglobulin, in 103 Chinese welding workers was significantly increased after exposure to 5~86 μg/m^3^ metal fumes in the breathing zone^5^. Hambach and colleagues (2013) found that exposure to metal fumes increased renal intestinal alkaline phosphatase expression and oxidative stress in workers^6^. Pulmonary exposure to heavy metals, such as cadmium, could be an important factor in renal injury of workers. Shelley and colleagues (2012) showed a potential correlation of the glomerular filtration rate to urine metal levels in lead workers^7^. Together, epidemiological studies have attempted to link occupational exposure to nephrotoxicity, but using a comprehensive approach to establish the correlation is required to provide more evidence.

Metal fumes exist as aerosolized nanoparticles of 10~100 nm in aerodynamic diameter and are usually produced in high-level concentrations during industrial processes, such as welding and cutting galvanized sheet metal. Such nanoscale particles are capable of being translocated into the circulation and accumulate in different organs after inhalation^8^. Excretion of inhaled metal fumes may be dependent on the physicochemical characteristics of the metals^9^,^10^. Pesch and colleagues (2000) further pointed out that occupational exposure to metals and solvents may be nephrocarcinogenic^11^. Actual exposure to metal fumes depends on the conditions in the occupational setting. For example, the permissible limit for occupational exposure to zinc oxide fume in the United States is set at 5 mg/m^3^ respirable dust. Therefore, preventing disease occurrence from exposure to welding processes under current guidelines for metal fume exposure remains controversial. Investigating the human health effects of pulmonary exposure to particulate matter of <2.5 μm in aerodynamic diameter (PM_2.5_) and its toxicity to secondary target organs such as the kidneys is urgently needed.

The objectives of this study were: (1) to discover the underlying potential pathways in urine of office workers and welding workers before and after 1-week exposure to PM_2.5_ of welding fumes; (2) to detect early diagnostic biomarkers for renal injury [i.e., kidney injury molecule (KIM)-1 and neutrophil gelatinase-associated lipocalin (NGAL)] in those workers; and (3) to evaluate the urinary metal concentrations and their correlations to KIM-1 and NGAL.

## Results

### Study population and exposure assessment

**Figure 1:**
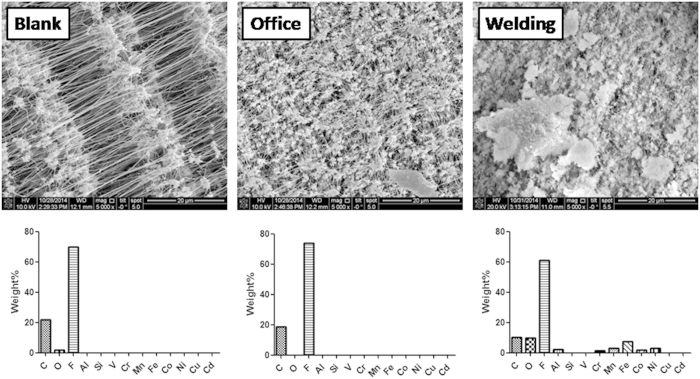
Physicochemical analyses of particles collected from the office and welding place. The particles were collected using two Micro-Orifice Uniform Deposit Impactors (MOUDI; MSP Inc., USA). The samples in the size between 1 μm and 1.8 μm were analysed using SEM and EDX. C, O, F, Al, Si, V, Cr, Mn, Fe, Co, Ni, Cu and Cd were determined in the blank, office and welding samples.

Sixty-six welding workers (melting metal processes) and 12 office workers were enrolled. Detailed baseline characteristics of the 78 subjects in the study population are presented in Table 1. The ages of welding workers and office workers were 51.0 ± 9.7 and 48.2 ± 15.3 years, respectively. All of the welding workers were men, but only 8% of the office worker group were men. Their body mass index (BMI) ranged 23.4~23.9 kg/m^2^. There were 14% current smokers in the welding worker group, whereas 8% of office workers were current smokers. Those who consumed alcohol were 27% of welding group and 33% of the office group. The 8-h mean PM_2.5_ concentrations were measured for welding workers and office workers, and were 50.3 ± 32.8 and 27.4 ± 16.2 μg/m^3^, respectively. The 8-h mean temperature and humidity were 24.1~27.5 °C and 63.1%~65.9%, respectively. The employment durations for the office workers and welding workers in the present study were 23.9 ± 16.3 years and 30 ± 12.5 years, respectively. The SEM-EDX results showed that C, O and F (from the filter material) were mainly observed in the blank filter and the office sample (Fig. 1). Additionally, Al (2.2%), Cr (1.3%), Mn (2.9%), Fe (7.4%), Co (1.8%) and Ni (3.1%) were detected in the welding samples.

### Proteomic profiling of the urine

There were 34 proteins identified in urine samples, among which 25 proteins were quantified for the four iTRAQ-labeled samples. iTRAQ ratios were obtained by the intensities of 115/114, 116/114, and 117/114, and protein ratios contributed by at least two peptide ratios are listed in Table 2. We observed that the lowest downregulated proteins in the post-exposure office workers were the AMBP protein (with a ratio of 0.706), the collagen alpha-2(I) chain (ratio of 0.661), and secretogranin-1 (ratio of 0.549), whereas the highest upregulated proteins were serum albumin (ratio of 1.376), kininogen-1 (ratio of 1.389), and RRP12-like protein (ratio of 1.509). The three lowest downregulated proteins in the pre-exposure welding workers were osteopontin (ratio of 0.428), secretogranin-1 (ratio of 0.491), and uromodulin (ratio of 0.438), whereas the three highest upregulated proteins were immunoglobulin (Ig) lambda-2 chain C regions (ratio of 2.308), basement membrane-specific heparan sulfate proteoglycan core protein (ratio of 1.824), and prostaglandin-H2 D-isomerase (ratio of 2.196). Notably, we observed that some of the down- and upregulated proteins in the post-exposure welding workers were similar to the pre-exposure welding group such as osteopontin (ratio of 0.761), Ig lambda-2 chain C regions (ratio of 2.177), and uromodulin (ratio of 0.559). Phosphoinositide-3-kinase-interacting protein 1 (ratio of 0.375), collagen alpha-1(I) chain (ratio of 3.034) and RRP12-like protein (ratio of 3.702) were only significantly expressed in welding workers.

### Pathways

**Figure 2:**
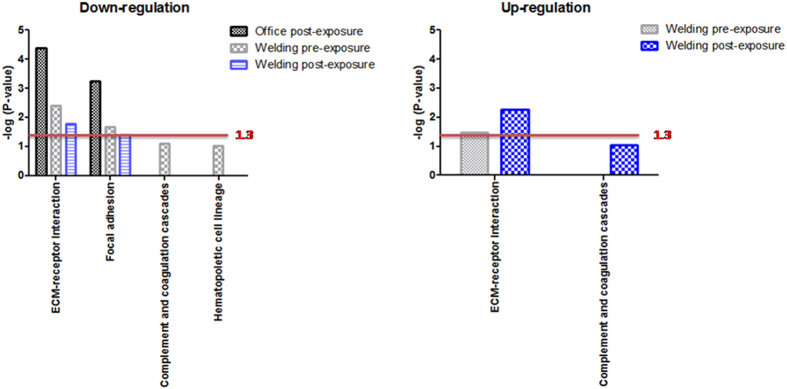
Pathways associated with the downregulation and upregulation of protein responses in post-exposure office workers, pre-exposure welding workers, and post-exposure welding workers were determined using a DAVID analysis. An enhanced score [−log(*p* value)] of ≥1.3 threshold (red line) was considered significant.

The DAVID analysis was conducted on up- and downregulated proteins in the post-exposure office workers, pre-exposure welding workers, and post-exposure welding workers to understand their pathways (Fig. 2). The extracellular matrix (ECM)-receptor interaction, focal adhesion, complement, and coagulation cascades and hematopoietic cell lineage were pathways identified among downregulated proteins. We found that only the ECM-receptor interaction and focal adhesion were significant pathways involved in downregulated proteins among the three groups, especially the ECM-receptor interaction (with enhanced scores of 4.37 for post-exposure office workers, of 2.41 for pre-exposure welding workers, and of 1.78 for post-exposure welding workers). There were two pathways identified among upregulated proteins in the pre-exposure welding workers and post-exposure welding workers; no pathways were found in the post-exposure office workers based on the identified proteins (Fig. 2). Only the ECM-receptor interaction was a significant pathway among upregulated proteins (with enhanced scores of 1.48 for pre-exposure welding workers and of 2.27 for post-exposure welding workers). Together, the ECM-receptor interaction was an important pathway in response to welding fume exposure, the expression of which was decreased in downregulated proteins and increased in upregulated proteins.

### Urinary KIM-1 and NGAL

**Figure 3:**
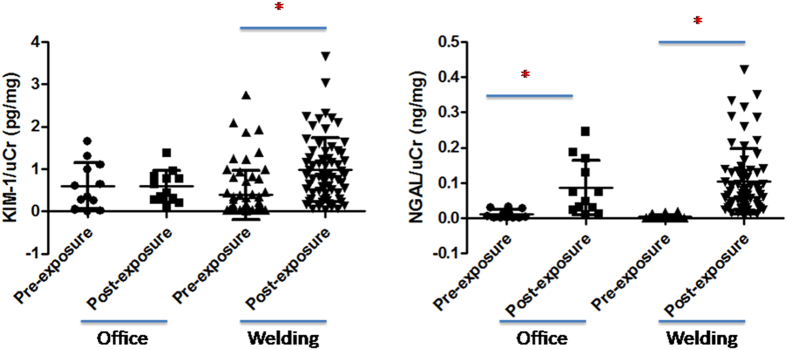
Urinary kidney injury molecule (KIM)-1 and neutrophil gelatinase-associated lipocalin (NGAL) levels after adjustment by urinary creatinine (uCr) in pre- and post-exposure office workers and welding workers. NGAL levels (adjusted by uCr) in post-exposure office workers and post-exposure welding workers were significantly higher than those in the pre-exposure groups, respectively (*p < 0.05).

Two ECM-receptor interaction-related markers, KIM-1 and NGAL, were used in this study. Levels of KIM-1 (adjusted by uCr) were significantly increased in post-exposure welding workers compared to pre-exposure welding workers (p < 0.05) (Fig. 3), but no difference was observed between pre-exposure and post-exposure office workers. NGAL levels (adjusted by uCr) in post-exposure office workers and post-exposure welding workers were significantly higher than those in the pre-exposure groups (p < 0.05), respectively (Fig. 3).

### Urinary metals

**Figure 4:**
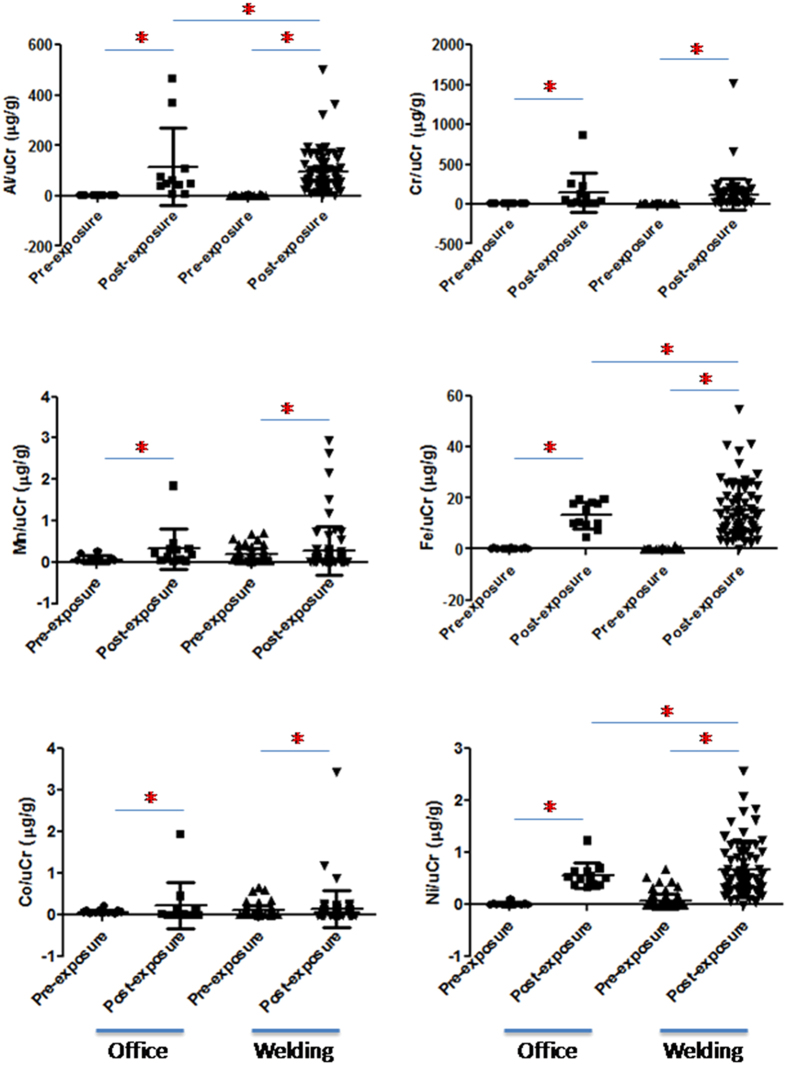
Urinary Al, Cr, Mn, Fe, Co, and Ni levels after adjustment by urinary creatinine (uCr) in pre-exposure office workers, post-exposure office workers, pre-exposure welding workers, and post-exposure welding workers. Al, Cr, Mn, Fe, Co, and Ni levels had significantly increased in post-exposure office workers and post-exposure welding workers compared to their pre-exposure levels. (*p < 0.05).

Urinary Al, Cr, Mn, Fe, Co, and Ni levels are shown in Fig. 4 after being adjusted by uCr in pre-exposure office workers, post-exposure office workers, pre-exposure welding workers, and post-exposure welding workers. We found that urinary Al, Cr, Mn, Fe, Co, and Ni levels were significantly increased in post-exposure office workers and post-exposure welding workers compared to their respective pre-exposure levels (p < 0.05). In particular, Al, Fe, and Ni, were higher in post-exposure welding workers than in post-exposure office workers (p < 0.05).

### Associations of urinary metals with KIM-1 and NGAL

Correlations of KIM-1 and NGAL with Al, Cr, Mn, Fe, Co, and Ni for welding workers and office workers were determined (Table 3). KIM-1 was associated with Al (r = 0.424, p < 0.001), Cr (r = 0.363, p < 0.001), Fe (r = 0.301, p < 0.001), and Ni (r = 0.223, p < 0.01), whereas NGAL was associated with Al (r = 0.737, p < 0.001), Cr (r = 0.705, p < 0.001), Mn (r = 0.379, p < 0.001), Fe (r = 0.709, p < 0.001), and Ni (r = 0.657, p < 0.001). Urinary NGAL levels had higher associations with urinary Al, Cr, Fe, and Ni than did urinary KIM-1 levels.

## Discussion

Pulmonary exposure to high-level concentrations of metal fumes observed in previous studies resulted in symptomatic, physiologic, and hematologic effects^3^,^12^. However, nephrotoxicity induced by metal fumes in welding workers remains unclear. The present study discovered differences in urinary protein expressions in workers who were exposed to low (27.4 μg/m^3^) and high levels of PM_2.5_ (50.3 μg/m^3^) during the study period. Three major findings are reported in the present study: (1) the ECM-receptor interaction was the most important pathway in response to welding fume exposure, (2) KIM-1 and NGAL were significantly increased post-exposure to welding fumes, and (3) NGAL was significantly associated with urinary Al, Cr, Fe, and Ni levels.

The relevant guideline for metal fume exposure in occupational environments lists a 5 mg/m^3^ time-weighted average (TLV) for an 8-h PM_10_ exposure^13^. In the present study, welding workers and office workers were exposed to 8-h mean PM_2.5_ levels of 50.3 and 27.4 μg/m^3^, respectively, which means the PM_2.5_ mass concentrations were significantly lower than the metal fume guideline. Hartmann and colleagues found that the 6-h mean concentration of welding fumes emitted from metal inert gas welding processes was 2.5 mg/m^3^^14^. Ding and colleagues showed that metal fume levels in a welder’s breathing zone ranged 5~86 μg/m^3^. Variations in metal fume concentrations between our and previous studies could have resulted from different conditions of the occupational environments (e.g., open, semi-open, and closed areas) and the welding processes. Also, the size fractions of metal fumes determined in our study and previous reports differed. In the present study, PM_2.5_ was measured for the personal exposure assessment. Particulate matter of <10 μm in aerodynamic diameter consists of thoracic particles, which can readily penetrate and deposit in the tracheobronchial tree^15^, whereas PM_2.5_ bypasses the upper airways and is deposited in lower and distal lung environments^16^,^17^. Nanoparticles (of  <100 nm in aerodynamic diameter) can be translocated into the circulatory system and enter different organs^8^,^18^. A previous report showed that inhaled zinc oxide nanoparticles were detected in end organs, such as the heart, after inhalation^19^, which could partially result from entrance of nanoparticles into the cardiopulmonary system due to their physiochemistry and their lack of phagocytic triggers^20^,^21^. Nanoparticles can be excreted by urine and feces after pulmonary exposure^10^. However, effects of excretion of metal fumes on the important filtration organ, the kidneys, remain unclear.

Proteomic techniques are widely applied to clinical and biological investigations^5^,^22^, and provide comprehensive information with which to understand biological networks. Proteomics can also reveal potentially deleterious health effects due to exposure in occupational and environmental settings^23^. To discover the pathways in response to exposure of metal fumes in urine, the iTRAQ-coupled LC-MS/MS approach was first used. We found that urinary phosphoinositide-3-kinase-interacting protein 1, collagen alpha-1(I) chain, and RRP12-like protein were only significantly expressed in welding workers after exposure. Phosphoinositide-3-kinase-interacting protein 1 is associated with regulation of multiple cellular processes as well as inflammatory reactions^24^. Previous studies showed that alteration of urinary levels of the collagen alpha-1(I) chain was associated with kidney diseases such as renal Fanconi syndrome^25^. To understand interactions among these significant downregulated and upregulated proteins in post-exposure office workers, pre-exposure welding workers, and post-exposure welding workers, DAVID analysis software was used to discover the underlying pathways among these proteins. The ECM-receptor interaction was a highly identical pathway in welding workers. The interaction between cells and the surrounding ECM is required for normal kidney development and function. The principal cellular receptors that mediate cell-ECM interactions are integrins, which are heterodimeric transmembrane glycoproteins^26^. Some metals were identified as being used for activation and synthesis of transporting proteins in the ECM^27^. Together, the ECM-receptor interaction may be a critical mechanism in response to metal fume exposure in welding workers.

KIM-1 and NGAL are related to ECM-receptor interaction pathways^28^, which are recognized to be clinical urinary biomarkers for renal injury. A previous study observed that urinary KIM-1 levels increased with exposure to trichloroethylene in Chinese factory workers^29^. However, associations of KIM-1 and NGAL with welding PM_2.5_ exposure remain unclear. Notably, we showed that welding workers after 1-week exposure to welding PM_2.5_ had significantly elevated KIM-1 and NGAL concentrations in their urine. KIM-1 is a member of the type I transmembrane glycoproteins, and is highly expressed and released by injured proximal tubular epithelial cells^28^. KIM-1 is persistently expressed in urine by tubular cells until injury recovery or repair, therefore, it is considered to be an ideal biomarker for tubular injury^30^. NGAL is a 25-kDa protein of the lipocalin family^31^. Urinary NGAL is produced by local distal tubule synthesis, whereas proximal tubule NGAL is derived from the circulating pool^32^. NGAL is rapidly expressed in response to renal tubular injury and different renal diseases such as acute kidney injury^28^, which is considered an early predictor of renal damage. Taking results for KIM-1 and NGAL together indicates that risk of renal tubular injury is increased in welding workers after metal fume exposure. More clinical evidence is required to investigate the effects of welding PM_2.5_ exposure on renal tubular injury in the future.

To determine the contributions of heavy metals to nephrotoxicity in welding workers, significantly elevated levels of Al, Cr, Mn, Fe, Co, and Ni in urine were further correlated with KIM-1 and NGAL. KIM-1 and NGAL were significantly related to Al, Cr, Fe, and Ni in the urine, particularly NGAL. The results suggested that NGAL may be a biomarker in the urine for occupational exposure to welding PM_2.5_, and elevated NGAL could imply an increased risk of renal tubular injury. Oxidative stress and inflammation may be the core mechanisms in response to exposure to Al, Cr, Fe, and Ni; however, mechanical experiments are necessary for confirmation in the future.

This study showed a nephrotoxic effect, as expressed by KIM-1 and NGAL, of exposure to PM_2.5_ in welding workers at concentrations below the metal fume guideline (5 mg/m^3^). The ECM-receptor interaction may play an important role in regulating nephrotoxicity that occurs due to welding fumes. However, we cannot eliminate the effects of PM_2.5_ emitted from welding processes in office workers. We observed that urinary NGAL and metals were higher in the post-exposure office workers. Because we have collected the particles in the office area during the study period, there was no significant metals in the particles observed using the SEM with EDX. We assume that smoking or outdoor exposure (welding particles) may be the possible contributors for the increase of NGAL and metals in the office workers’ urine. Personal exposure assessment is important to clarify this issue, which will be conducted in our future works. Heavy metal intake from food and drink may be other sources that were not measured in the present study. The sample size and gender distribution in the welding and office groups significantly differed. Other environmental factors such as ozone as well as physical workload should be considered in the future studies. Our findings provide additional evidence, for the first time, that exposure to heavy metals in PM_2.5_ increases the risk of renal injury in welding workers.

## Materials and Methods

### Ethics

The Ethics Committee of the Taipei Medical University-Joint Institutional Review Board approved the study protocol. The methods were carried out in accordance with the approved guidelines. All subjects received written and oral information prior to inclusion and provided informed consent.

### Study population and exposure assessment

This study was designed to investigate the association between personal exposure to welding fume PM_2.5_ with urinary biomarkers determined by proteomic approaches and urinary metals among study subjects from a shipyard located in southern Taiwan. Tungsten inert gas (TIG) welding was the main method in the company. There were 66 welding workers and 12 office workers recruited for this study. Exclusion criteria for participants were those who had cardiovascular or renal disease or a history of cardiovascular and renal disease. Urine samples from each subject were collected at the beginning (Monday; pre-exposure; baseline for 1-week exposure) and end of the work week (Friday; post-exposure; 1-week exposure). Personal exposure to PM_2.5_ was measured for each subject from 08:00 to 20:00 using a real-time dust monitor (DUST-check Portable Dust Monitor model 1.108, Grimm Labortechnik, Ainring, Germany) that measured and recorded 1-min mass concentrations of PM_2.5_, as well as temperature and relative humidity. A baseline screening questionnaire was administered to assess the age, sex, body-mass index (BMI), smoking, alcohol consumption, medications, and working characteristics.

### Characterization of particles

Two Micro-Orifice Uniform Deposit Impactors (MOUDI; MSP Inc., USA) were set up in the office and welding place with a constant flow rate of 30 l/min. The MOUDIs were used to collect particles ranging between 0.056 μm and 18 μm in mean diameter using 11 inertial-based cascade impactors^33^. Particles were collected onto 37 mm Teflon without greasing. An Inspect™ field emission-scanning electron microscope (FE-SEM; JEOL 2100, Jeol, Japan) and an energy-dispersive *X*-ray (EDX) microanalysis instrument were used to determine the physicochemistry of particles in the size between 1 μm and 1.8 μm collected from the office and welding areas, according to a previous report^19^. Briefly, the particles were investigated via adhesion to 12*-*mm carbon adhesive tabs on 13-mm aluminium SEM stubs. The SEM stubs were coated with platinum (Pt) with an average thickness of 10 nm using a sputter coater. FE-SEM images were obtained using the following conditions: an accelerating voltage of 10 kV and a 5 spot size. Elemental analysis was conducted using the EDX Genesis Microanalysis System.

### Urinary sample preparation and isobaric tags for relative and absolute quantitation (iTRAQ) labeling

To investigate the potential pathways of PM_2.5_ exposure on urinary protein expressions in welding worker, three welding workers and three office workers were randomly selected from the study population. Urinary samples from the welding workers (pre-exposure and post-exposure) and office workers (pre-exposure and post-exposure) were pooled together into four groups to minimize individual variations in proteomic studies^34^: a pre-exposure welding group, a post-exposure welding group, a pre-exposure office group, and a post-exposure office group. Urine samples were precipitated with acetone at −20 °C for 2 h and then re-dissolved in 0.5 M triethylammonium bicarbonate (TEAB). Subsequent reduction, alkylation, digestion, and iTRAQ labeling (Applied Biosystems, Grand Island, NY, USA) were performed according to the manufacturer’s protocol. Briefly, samples were reduced in 5 mM Tris-(2-carboxyethyl)phosphine (TCEP), and cysteine groups were blocked by a solution of 10 mM methyl methanethiosulfonate (MMTS). Samples were digested with 10 μg trypsin and then labeled with the iTRAQ tag (114 for the pre-exposure office group, 115 for the post-exposure office group, 116 for the pre-exposure welding group, and 117 for the post-exposure welding group). Samples were combined, dried using a speedvac, re-dissolved in 50 μl of 5% acetone in 0.1% formic acid, and subjected to further analysis.

### Liquid chromatography-tandem mass spectrometry (LC-MS/MS)

iTRAQ-labeled samples were combined and analyzed using a nano LC-Quadruple Orbitrap equipped with high energy collision dissociation (HCD) technology^35^. Briefly, samples were analyzed using a Q Exactive mass spectrometer (Thermo Fisher Scientific, Bremen, Germany) coupled with an UltiMate 3000 RSLC system (Dionex, Sunnyvale, USA). The HCD fragmentation mode was used to generate MS and MS/MS spectra. LC separation was performed using C18 columns (75 μm × 150 mm, 2 μm, 100 Å; Acclaim PepMap RSLC, Dionex., USA) using the following conditions for the full MS scans: m/z 380~2000, m/z 380~600, m/z 600~800, m/z 800~1200, and m/z 1200~2000. The ten most intense ions were subjected to fragmentation for the MS/MS spectra. Raw data were processed into peak lists by searching the Proteome Discoverer 1.4 for Mascot database using search parameters including variable modifications for deamidation (NQ) and oxidation (M) and fixed modifications for methylthio (C), iTRAQ 4plex (N-term), and iTRAQ 4plex (K). The maximum mass tolerance was set to 10 ppm for precursor ions and to 0.05 Da for fragmented ions. Only peptides with Mascot scores of  >30 at the 95% confidence level were further considered. The criteria used for selection of differentially expressed proteins included the following: at least two unique high-scoring peptides in the protein, *p* < 0.05 across two independent iTRAQ experiments, and a protein ratio of  >1.3 or  <0.77^36^.

### Protein functional analyses

Upregulated and downregulated proteins in the 115/114 (post-exposure office group to pre-exposure office group), 116/114 (pre-exposure welding group to pre-exposure office group), and 117/114 groups (post-exposure welding group to pre-exposure office group) were subjected to functional pathway analyses using the Database for Annotation, Visualization and Integrated Discovery (DAVID) gene functional analytical tools (http://david.abcc.ncifcrf.gov/) to determine the corresponding pathways^37^,^38^. For the DAVID analysis, an enhanced score of  ≥1.3, set as the threshold, was considered significant, as described in a previous report^39^.

### Urinary KIM-1 and NGAL

An enzyme-linked immunosorbent assay (ELISA) was used to determine urinary KIM-1 (Enzo, Farmingdale, NY, USA) and NGAL (R&D, Minneapolis, MN, USA), according to the manufacturer’s instructions. Levels of KIM-1 and NGAL were adjusted by urinary creatinine (uCr).

### Urinary metal concentrations

Urine samples were digested using concentrated nitric acid (Fisher Scientific, USA) in a MARS 5 microwave system (CEM, USA) in CEM advanced Teflon-lined composite vessels^19^, followed by 0.45-μm polyvinylidene difluoride filtration (ChromTech, USA). Nitric acid and deionized (>18 MΩ) water were added to the samples for a final concentration of 5% nitric acid. Inductively coupled plasma-mass spectrometry (ICP-MS; Agilent 7500, USA) was used to determine the following seven metal concentrations in urinary samples: aluminum (Al), chromium (Cr), manganese (Mn), iron (Fe), cobalt (Co), and nickel (Ni). Deionized water blanks were analyzed to detect any contamination during the analytical process. A solution of a certified rock standard (BCR1) was used to determine the accuracy of the analyses. The relative percentage difference was <10%.

### Statistical analysis

Data are expressed as the mean ± standard deviation (SD). The Shapiro-Wilk test was used to test for normality. Chi-square test was used to determine the difference in percentage of male, smoking and alcohol consumption between groups. For comparisons between two groups, the Student’s t-test was used. For comparisons among multiple values, a one-way analysis of variance (ANOVA) with Tukey’s post-hoc test was used. Spearman’s correlation was used to assess associations of KIM-1 and NGAL with metals in the urine. All of the statistical analyses in this study were performed with SPSS 15.0 software (SPSS, New York, NY, USA). The level of significance for all of the statistical analyses was set to *p* < 0.05.

## Additional Information

**How to cite this article**: Chuang, K.-J. *et al.* Urinary neutrophil gelatinase-associated lipocalin is associated with heavy metal exposure in welding workers. *Sci. Rep.* **5**, 18048; doi: 10.1038/srep18048 (2015).

## Figures and Tables

**Table 1:** Demographic characteristics, exposure to fine particulate matter (PM_2.5_), and meteorological conditions (temperature and humidity) of 78 study subjects (66 welding workers and 12 office workers).

	Welding workers (N = 66)	Office workers (N = 12)	P-value
Male (%)	100	8	0.018*
Age, mean ± SD (years)	51.0 ± 9.7	48.2 ± 15.3	0.148
Body-mass index, mean ± SD (kg/m^2^)	23.9 ± 3.1	23.4 ± 2.5	0.892
Smoking (%)	14	8	0.354
Alcohol consumption (%)	27	33	0.487
8-h mean PM_2.5_, mean ± SD (μg/m^3^)	50.3 ± 32.8	27.4 ± 16.2	0.019*
8-h mean temperature, mean ± SD (°C)	27.5 ± 2.6	24.1 ± 2.1	0.116
8-h mean humidity, mean ± SD (%)	65.9 ± 5.1	63.1 ± 3.3	0.083

^*^P-value < 0.05 for comparison between welding and office workers.

**Table 2:** Proteomic profiling in urine among post-exposure office workers (115/114), pre-exposure welding workers (116/114) and post-exposure welding workers (117/114) using iTRAQ coupled with LC-MS/MS.

Accession no.	Protein description	Protein score	MW (kDa)	Sequence covered (%)	Protein PI	Office workers (post-exposure)	Welding workers (pre-exposure)	Welding workers (post-exposure)
						115/114	116/114	117/114
A1AG1	Alpha-1-acid glycoprotein 1	44	25842	7.5	4.93	1.366	1.431	1.160
ALBU	Serum albumin	3812	79721	47.8	5.92	1.376	1.190	1.264
AMBP	Protein AMBP	1305	42448	36.4	5.95	0.706	1.254	0.953
APOD	Apolipoprotein D	60	23221	10.1	5.06	1.116	0.872	0.775
CD44	CD44 antigen	80	84927	1.1	5.13	1.226	0.812	1.117
CD59	CD59 glycoprotein	179	15826	6.3	6.02	0.866	0.938	1.512
CO1A1	Collagen alpha-1(I) chain	55	148042	1.2	5.6	0.838	1.043	3.034
CO1A2	Collagen alpha-2(I) chain	58	136999	1.1	9.08	0.661	0.801	0.787
FLNA	Filamin-A OS = Homo sapiens	66	305972	0.3	5.7	0.825	0.923	1.380
IGHG2	Ig gamma-2 chain C region	101	39986	2.5	7.66	1.127	1.184	1.864
IGKC	Ig kappa chain C region	940	13037	80.2	5.58	0.825	1.658	1.443
KNG1	Kininogen-1	161	80279	8.5	6.34	1.389	0.739	1.155
LAC2	Ig lambda-2 chain C regions	325	12721	67	6.92	1.105	2.308	2.177
OSTP	Osteopontin	37	38421	5.1	4.37	0.964	0.428	0.761
P3IP1	Phosphoinositide-3-kinase-interacting protein 1	101	30039	4.2	4.92	1.054	0.762	0.375
PGBM	Basement membrane-specific heparan sulfate proteoglycan core protein	76	487121	0.5	6.06	0.922	1.824	1.375
PIGR	Polymeric immunoglobulin receptor	105	90394	4.7	5.58	1.183	0.933	1.609
PTGDS	Prostaglandin-H2 D-isomerase	601	22784	24.2	7.66	1.194	2.196	2.159
RNAS1	Ribonuclease pancreatic	50	19442	20.5	9.1	—	1.453	—
RRP12	RRP12-like protein	43	158739	0.5	8.97	1.509	0.952	3.702
SCG1	Secretogranin-1	107	85238	4.1	5.02	0.549	0.491	0.925
UROM	Uromodulin	2777	74372	22.7	5.05	0.87	0.438	0.559
YIPF3	Protein YIPF3	196	39703	2.9	5.47	0.927	0.793	1.754
ZA2G	Zinc-alpha-2-glycoprotein	48	37447	7.7	5.71	1.203	1.340	1.806

– Not available. The iTRAQ ratios were obtained by the intensity of 115/114, 116/114 and 117/114, and the protein ratios contributed by at least two peptide ratios.

**Table 3:** Correlations between KIM-1 and NGAL with Al, Cr, Mn, Fe, Co and Ni for the welding workers and office workers.

	KIM-1	NGAL
Al	0.424**	0.737**
Cr	0.363**	0.705**
Mn	0.104	0.379**
Fe	0.301**	0.709**
Co	0.028	0.080
Ni	0.223*	0.657**

Urinary NGAL levels had higher association with urinary Al, Cr, Fe and Ni than KIM-1.

^*^p < 0.01

^**^p < 0.001.
